# Endosomes as Signaling Platforms for IL-6 Family Cytokine Receptors

**DOI:** 10.3389/fcell.2021.688314

**Published:** 2021-06-01

**Authors:** Dirk Schmidt-Arras, Stefan Rose-John

**Affiliations:** Institute of Biochemistry, Christian-Albrechts-University Kiel, Kiel, Germany

**Keywords:** IL-6, IL-11, GP130, cytokine, endosome, signal transduction, inflammation

## Abstract

Interleukin-6 (IL-6) is the name-giving cytokine of a family of eleven members, including IL-6, CNTF, LIF, and IL-27. IL-6 was first recognized as a B-cell stimulating factor but we now know that the cytokine plays a pivotal role in the orchestration of inflammatory processes as well as in inflammation associated cancer. Moreover, IL-6 is involved in metabolic regulation and it has been shown to be involved in major neural activities such as neuroprotection, which can help to repair and to reduce brain damage. Receptor complexes of all members formed at the plasma membrane contain one or two molecules of the signaling receptor subunit GP130 and the mechanisms of signal transduction are well understood. IL-6 type cytokines can also signal from endomembranes, in particular the endosome, and situations have been reported in which endocytosis of receptor complexes are a prerequisite of intracellular signaling. Moreover, pathogenic GP130 variants were shown to interfere with spatial activation of downstream signals. We here summarize the molecular mechanisms underlying spatial regulation of IL-6 family cytokine signaling and discuss its relevance for pathogenic processes.

## Introduction

Interleukin-6 (IL-6) – together with IL-1β and TNFα – is one of the major inflammatory cytokines, which is elevated in most if not all inflammatory states and has also been recognized as a frequent growth factor in many cancers (Grivennikov et al., 2009; Lesina et al., 2011; Garbers et al., 2018; Jones and Jenkins, 2018). IL-6 activity is also an important target of therapy in autoimmune diseases (Kang et al., 2019). The biology of IL-6, which has been cloned 35 years ago (Hirano et al., 1986), is complex and not completely understood (Rose-John, 2018).

IL-6 was originally identified and cloned as a B-cell stimulating factor (Hirano et al., 1986) but it soon turned out that it was identical with hepatocyte stimulating factor (Gauldie et al., 1987), hybridoma growth factor (Brakenhoff et al., 1987) and human interferon beta-2 (Zilberstein et al., 1986), pointing to a pleiotropic spectrum of activities. Now we know that IL-6 plays a prominent role in many inflammatory states and cancer. Moreover, IL-6 has prominent metabolic functions (Wallenius et al., 2002) and is an important factor in neural development (Gadient and Otten, 1994).

**FIGURE 1:**
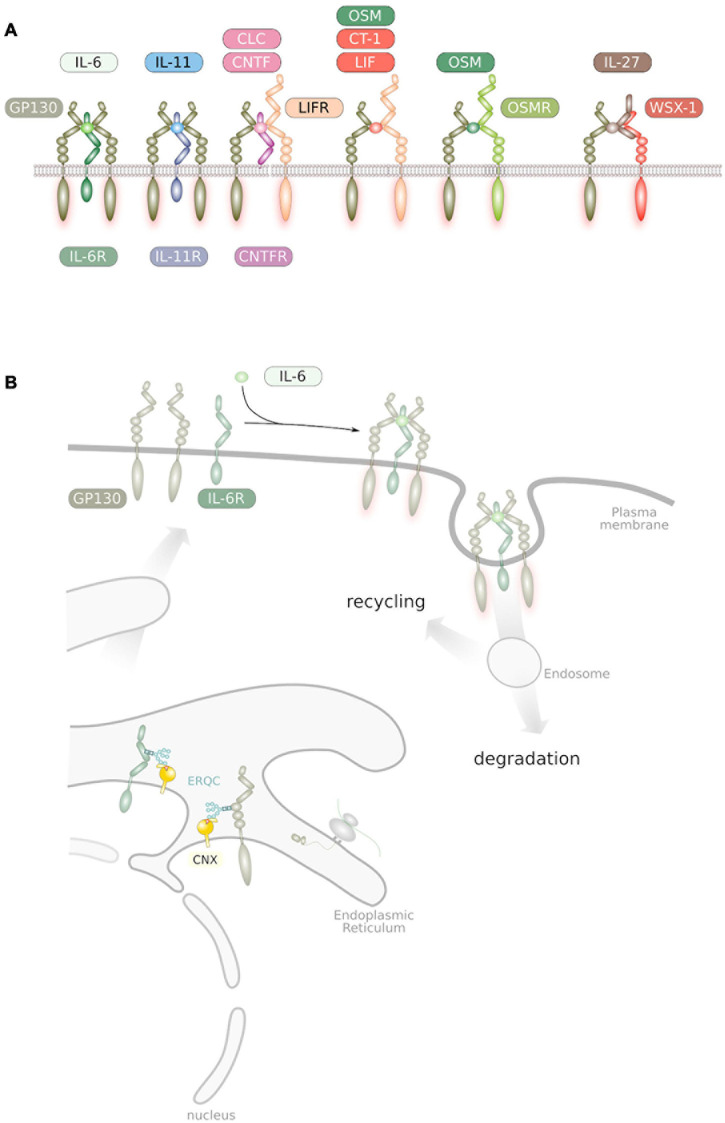
**(A)** Overview of IL-6 family cytokine receptor complexes. GP130, LIFR, OSMR, and IL-27Rα/WSX-1 are the only receptor subunits of the family that interact with members of the Janus kinase family and are therefore competent for signal transduction (Schmidt-Arras et al., 2021). **(B)** Trafficking of IL-6 family cytokine receptors. Receptors get synthesized into the endoplasmic reticulum and subsequently glycosylated at Asn residues. These glycans are essential during passage through the ER quality control. Ligand binding at the plasma membrane causes receptor homo/heterodimerization. Internalized receptors are either subjected to degradation or to recycling back to the plasma membrane.

Human IL-6 is a four helical glycosylated protein of 184 amino acids (Reif et al., 2021), which shares an overall structural homology with many other cytokines (Spangler et al., 2015). On target cells, IL-6 binds to a membrane-bound IL-6 receptor (IL-6R) and the complex of IL-6 and IL-6R associates with a second receptor subunit called GP130 leading to an onset of intracellular signaling via the janus kinase (JAK)/signal transducer and activator of transcription (STAT), phosphoinositide-3 kinase (PI3K)/AKT kinase and protein tyrosine phosphatase non-receptor type (PTPN) 11/SHP2/mitogen activated protein kinase (MAPK) pathway (Schaper and Rose-John, 2015; Jones and Jenkins, 2018; Rose-John, 2018). Interestingly, GP130 has been recognized to be a subunit of the receptor complexes of IL-11, IL-27, leukemia inhibitory factor (LIF), ciliary neurotrophic factor (CNTF), cardiotrophin like cytokine (CLC), oncostatin M (OSM) and cardiotrophin-1 (CT-1), which together form the IL-6 family of cytokines (Figure 1A; Schaper and Rose-John, 2015; Jones and Jenkins, 2018; Rose-John, 2018).

It has been shown that the IL-6R is readily shed from the cell surface of human and murine cells (Müllberg et al., 1993) and is found in human blood (Riethmueller et al., 2017). Interestingly, in humans but not in mice, the soluble IL-6R (sIL-6R) can also be generated by translation from an alternatively spliced mRNA (Lust et al., 1992), although this mechanism accounts only for about 10% of sIL-6R that is found in human blood (Riethmueller et al., 2017). The sIL-6R binds IL-6 (Müllberg et al., 1993) and the complex of IL-6 and sIL-6R stimulates cells, which only express GP130 but no IL-6R (Mackiewicz et al., 1992). Except on mature granulocytes (Wilkinson et al., 2018), *IL6ST*, encoding GP130, is expressed on all cells of the body. However, expression levels vary. High expression levels were found in the liver, in particular hepatic stellate cells, placenta, breast and lymph node. In leukocytes, *IL6ST* is in particular expressed in T-cells (Fagerberg et al., 2014; Uhlén et al., 2015). In contrast, *IL6R* expression is very low in most of the tissues and elevated expression has been found in both types of alveolar cells, hepatocytes, some leukocytes, such as granulocytes, and in the skeletal muscle (Rose-John et al., 1990; Oberg et al., 2006). The ratio of GP130 and alpha receptor such as IL-6R therefore dictates cytokine responsiveness of an individual cell. However, expression data have to be handled with caution as expression levels vary depending on the deposited dataset and expression of *IL6ST* and *IL6R* should be validated experimentally in the tissue of interest.

Cells without IL-6R expression are completely unresponsive to IL-6 (Mackiewicz et al., 1992). The mode of signaling via the sIL-6R significantly enlarges the spectrum of target cells of IL-6 and has been called IL-6 trans-signaling (Rose-John and Heinrich, 1994). Similarly, it has been demonstrated *in vitro* that IL-11 bound to the sIL-11R can stimulate GP130 expressing cells although the *in vivo* relevance of this process has not yet been elucidated (Lokau et al., 2016). Interestingly, Human Herpes Virus 8 encodes a protein, which shows 25% sequence identity with human IL-6 (Neipel et al., 1997). This viral IL-6 (vIL-6) protein, a soluble protein without transmembrane domain, was shown to directly bind to GP130 without being presented by the human IL-6R (Chow et al., 2001). Therefore, the vIL-6 protein stimulates cells in the absence of IL-6R and therefore shows the same spectrum of target cells as the IL-6/sIL-6R complex via trans-signaling (Molden et al., 1997; Müllberg et al., 2000). In addition to the sIL-6R, which is found in the blood of healthy individuals at concentrations of about 40–80 ng/ml, soluble forms of gp130 are found in the blood at levels of about 400 ng/ml (Garbers et al., 2018; Rose-John, 2018). It is believed that sIL-6R and sgp130 form a buffer for IL-6, which in healthy volunteers is found at 1–5 pg/ml but which rises during inflammatory states by several 100- to 1000-fold (Garbers et al., 2018; Rose-John, 2018).

For a long time, activation of signal proteins by receptor complexes at the plasma membrane were thought to be the only source of downstream signaling. In this monolithic view, internalization of receptor complexes has been solely considered to terminate receptor signaling. However, emerging data suggest that receptor complexes internalized into endosomes can serve as signaling platforms that support sustained intracellular signaling, potentially even with altered signal quality. Here, we summarize current knowledge and discuss the importance of endomembranes, in particular endosomes, for the signal transduction of IL-6 family cytokines.

## The Interleukin-6 Family of Cytokines

The IL-6 family of cytokines is defined by the presence of GP130 in their cognate receptor complexes (Figure 1A). IL-6 and IL-11 bind to their specific IL-6R and IL-11R receptor subunits and subsequently associate with a homodimer of GP130 (Figure 1A; Kishimoto, 2005). The cytokines CNTF and CLC interact with the CNTF-R and signal via a heterodimer formed by GP130 and the related protein LIF-R (Kishimoto, 2005). OSM directly binds to GP130 leading to heterodimer formation with LIF-R whereas LIF directly binds to the LIF-R, which heterodimerizes with GP130 (Kishimoto, 2005). OSM can also bind to an alternative receptor complex which is formed by GP130 and the OSM-R (Mosley et al., 1996). IL-27 is a dimeric cytokine formed by the four-helical protein p28 and the soluble cytokine receptor-like protein EBI3, which binds to a heterodimer formed of GP130 and WSX-1 (Pflanz et al., 2002; Figure 1A).

Several designer proteins have been generated to study the biology of IL-6 and the relevance of GP130 signaling. Hyper-IL-6 is a fusion protein of sIL-6R covalently connected to the NH_2_ terminus of IL-6 by a flexible peptide linker (Fischer et al., 1997). Hyper-IL-6 mimics IL-6 trans-signaling and was used to differentiate between classic- and trans-signaling. Hyper-IL-6 but not IL-6 alone strongly stimulated the expansion of hematopoietic stem cells (Audet et al., 2001) and the survival of sympathetic neurons (März et al., 1998). Smooth muscle cells (Klouche et al., 1999), endothelial cells (Romano et al., 1997), and embryonic stem cells (Humphrey et al., 2004) are only responsive to IL-6 in the presence of sIL-6R. Moreover, it was shown that liver regeneration, which is largely dependent on IL-6 (Cressman et al., 1996) was significantly accelerated in the presence of sIL-6R (Galun et al., 2000; Peters et al., 2000).

While Hyper-IL-6 demonstrated the enormous *in vitro* and *in vivo* potential of IL-6 trans-signaling, it did not prove that this signaling mode actually occurred *in vivo*. Therefore a second designer protein was generated, which was called soluble gp130Fc (sgp130Fc) (Jostock et al., 2001). The sgp130Fc protein consists of the entire extracellular portion of GP130 fused to the Fc portion of a human IgG1 antibody (Jostock et al., 2001). It turned out that sgp130Fc exclusively blocked IL-6 trans-signaling without affecting classic IL-6 signaling via the membrane-bound IL-6R (Jostock et al., 2001). The reason for this specificity was the fact that GP130 shows no measurable affinity for the separate proteins IL-6 or IL-6R. It only binds the complex of IL-6 bound to the IL-6R (Jostock et al., 2001). Therefore, stimulation of cells, which express IL-6R with IL-6 will not be affected by sgp130Fc since IL-6 bound to the membrane-bound IL-6R immediately associates with membrane-bound GP130 and sgp130Fc has no access to the receptor-bound IL-6. In contrast, the complex of IL-6/sIL-6R in solution can just as well bind to membrane-bound GP130 as to sgp130Fc. In the presence of a molar excess of sgp130Fc, IL-6 trans-signaling will be completely blocked (Jostock et al., 2001).

It has been shown that IL-6 trans-signaling is involved in many pathophysiologic states including autoimmunity and cancer (Jones et al., 2011). Recent work demonstrated that specific blockade of IL-6 trans-signaling prevented high-fat diet induced adipose tissue macrophage accumulation (Kraakman et al., 2015) and blocked IL-6 mediated neurodegeneration in a transgenic animal model (Campbell et al., 2014). Recently it was found that IL-6 trans-signaling strongly stimulated repopulation of microglia in the mammalian brain and thereby aided to repair cognitive deficits from brain injury (Willis et al., 2020).

Inhibition of IL-6 activity by neutralizing antibodies to the IL-6R has been approved in many countries for the treatment of autoimmune diseases (Tanaka et al., 2014; Kang et al., 2019). The neutralizing antibodies tocilizumab and sarilumab have been approved for the treatment of patients with autoimmune diseases such as Rheumatoid Arthritis (Garbers et al., 2018; Jones and Jenkins, 2018; Kang et al., 2019). Both antibodies block the binding of the ligand IL-6 to the IL-6R (Garbers et al., 2018; Jones and Jenkins, 2018). This helps to avoid a problem seen with neutralizing IL-6 antibodies, which led to enormous accumulation of IL-6 bound to the antibody in the circulation of patients (Lu et al., 1992). In contrast, treatment with neutralizing IL-6R antibodies resulted in only slight elevation of serum IL-6 levels, which were explained by an inhibition of IL-6 internalization via the membrane-bound IL-6R (Nishimoto et al., 2008). However, IL-6R internalization and degradation rates were not altered by the binding of tocilizumab *in vitro* (Fujimoto et al., 2015). Blockade of IL-6 biologic activity with the help of IL-6R neutralizing antibodies has been approved in many countries. The blockade of IL-6 activity is highly successful and has been shown to be equivalent or superior to the blockade of the cytokine TNFα (Gabay et al., 2013; Burmester et al., 2017). Interestingly, we have shown that specific blockade of IL-6 trans-signaling was as effective as the blockade of global IL-6 activity by a neutralizing antibody indicating that IL-6 trans-signaling represents the pro-inflammatory IL-6 activity (Scheller et al., 2011) whereas IL-6 signaling via the membrane bound IL-6R was rather protective e.g., in the case of bacterial infections (Sodenkamp et al., 2012; Hoge et al., 2013). Having shown that the sgp130Fc protein protected mice in models of inflammatory bowel disease (Atreya et al., 2000; Mitsuyama et al., 2006) we could recently demonstrate the efficacy of the sgp130Fc proteins in patients with Crohn’s disease and ulcerative colitis (Schreiber et al., 2021).

During inflammatory states, IL-6 is secreted by many cell types including myeloid cells, fibroblasts, endothelial cells and T-cells (Kang et al., 2020) and the response to IL-6 stimulation differs between cell types (Jones and Jenkins, 2018). In order to define the cell specific response to IL-6 we have generated a constitutively activated GP130 molecule, which was dimerized by a leucine zipper (Stuhlmann-Laeisz et al., 2006) and inserted this construct, which we termed LGP130, into the ROSA26 locus of mice (Scherger et al., 2019). This mouse model allows to activate GP130 signaling in a cell-autonomous manner in every selected cell type by breeding these mice to appropriate Cre-expressing transgenic mice (Haldar et al., 2007; Scherger et al., 2019). This mouse model allowed us recently to define the interplay of cell-autonomous, activated GP130 in hepatocytes and a systemic innate immune response, which was likely triggered by the hepatic GP130-induced expression of acute phase proteins such as serum amyloid A (Schumacher et al., 2021).

## The Life-Cycle of Il-6 Family Receptor Complexes

### Expression of IL-6 Receptor Proteins

Response to IL-6 family cytokines is largely determined by the expression of the corresponding receptor complex proteins and it was demonstrated that expression levels of *IL6ST*, encoding GP130, and *LIFR* are controlled by epigenetic mechanisms. Inhibition of histone H3 acetylation resulted in elevated expression of IL6ST and LIFR in certain cell types (Blanchard et al., 2002). The promoter region of *IL6ST* contains several transcription factor binding sites, including those for CCAAT/enhancer binding protein (C/EBP) β, SP1, STAT1/3 (O’Brien and Manolagas, 1997) and NFκB. It is therefore not surprising that *IL6ST* expression can be induced by several cytokines, including IL-1β, IL-6, IL-10, OSM, and IFNγ (Romas et al., 1996; O’Brien and Manolagas, 1997; Blanchard et al., 2001) that either induce STAT1, STAT3, or NFκB activity. Furthermore, the mitogen-activated protein kinase (MAPK) ERK2 was found to be associated with the *IL6ST* promoter and enhances *IL6ST* expression most likely via phosphorylation of the SP1 transcription factor (Bonito et al., 2014). In isolated murine mast cells, IL-10 strongly induced *IL6ST* expression and subsequently GP130 surface localization and in consequence sensitivity of mast cells toward WSX-1 (Traum et al., 2012). The designer cytokine HyperIL-6 is also able to robustly induce *IL6ST* expression and subsequent GP130 plasma membrane accumulation in isolated aortic smooth muscle cells (Klouche et al., 1999).

### Trafficking of Signal Transducing Receptors

Receptors for IL-6 family cytokines are type I transmembrane proteins. As such they are synthesized into the endoplasmic reticulum (ER), undergo N-linked glycosylation with high-mannose glycan structures and are subject of the ER quality control (Figure 1B; Caramelo and Parodi, 2015). Upon ER exit, glycan structures are modified within the Golgi before receptors traffic to the plasma membrane. Full maturation and trafficking of GP130 to the plasma membrane was demonstrated to occur within one to four hours, depending on the cellular system (Gerhartz et al., 1994; Wang and Fuller, 1995; Schmidt-Arras et al., 2014). GP130 becomes N-glycosylated at several asparagine residues within its extracellular domain (Moritz et al., 2001; Xu et al., 2010). The attached glycans most likely assist in GP130 folding as mutation of N-glycosylation sites largely resulted in localization of GP130 in a perinuclear compartment, most likely the endoplasmic reticulum (Waetzig et al., 2010). Consequently, deficient GP130 glycosylation induced either via mutagenesis or via pharmacological inhibition resulted in impaired cellular sensitivity toward IL-6 family cytokines (Waetzig et al., 2010; Matsuo et al., 2014).

**FIGURE 2:**
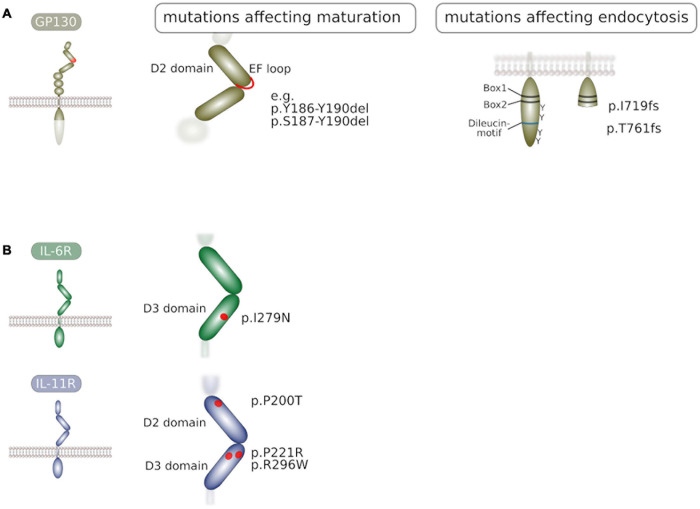
**(A)** Mutations in GP130 that interfere with receptor maturation or receptor endocytosis. Activating deletion mutations in GP130 were found in inflammatory hepatocellular adenoma and impair GP130 maturation. Frameshift mutations that lead to truncated GP130 molecules devoid tyrosine residues and internalization motif are found in patients. **(B)** Mutations in the α receptor subunits IL-6R and IL-11R that impair receptor maturation to the plasma membrane. Point mutations in IL-6R are found in patients with a novel immunodeficiency syndrome. Point mutations in IL-11R are found in patients suffering from craniosynostosis. Prolonged residence of these mutants within the ER quality control is likely.

In addition to glycosylation, trafficking of GP130 to the plasma membrane is also hampered by premature intracellular activity of GP130. Activating deletion mutations in *IL6ST* are found in patients suffering from inflammatory hepatocellular adenoma (IHCA). These deletions vary in size and cluster at the EF loop of domain 2 (Figure 2A) that is part of the cytokine-binding module of GP130 (Rebouissou et al., 2009; Schütt et al., 2013). The surface localization of these constitutively active GP130 variants is largely lowered (Rinis et al., 2014; Schmidt-Arras et al., 2014) due to prolonged association with the lectin-base chaperone calnexin within the ER quality control (Schmidt-Arras et al., 2014) that results in delayed GP130 maturation. Similar observations were made for oncogenic constitutively active receptor tyrosine kinases (Schmidt-Arras and Böhmer, 2020) and suggest that either altered ectodomain conformations or downstream signaling pathways modify receptor processing and trafficking to the plasma membrane.

Little is known about biosynthesis and trafficking of the signal-transducing IL-6 family receptor members LIFR and OSMR.

### Trafficking of Alpha Receptors

In polarized MDCK cells, the IL-6R is synthesized and transported to the plasma membrane within one hour. During the biosynthetic process, IL-6R becomes N-glycosylated at Asn residues within the extracellular domain (Gerhartz et al., 1994; Riethmueller et al., 2017). IL-6R glycosylation seems to be dispensable for ligand binding and trafficking to the plasma membrane (Riethmueller et al., 2017). In contrast, deletion of the N-terminal Ig-like domain strongly reduces plasma membrane localization of IL-6R most likely due to aberrant receptor maturation (Vollmer et al., 1999). Four hours after synthesis wildtype IL-6R becomes degraded independent of ligand-binding (Gerhartz et al., 1994; Flynn et al., 2021), suggesting that similar to GP130, internalization of IL-6R occurs independent of receptor activity. The short cytoplasmic domain was shown to mediate basolateral sorting in polarized MDCK cells and deletion of the cytoplasmic domain resulted in apical rerouting of IL-6R (Martens et al., 2000). In contrast, the close homolog, IL-11R is present at both, basolateral and apical sides in MDCK cells (Monhasery et al., 2016). Similar to IL-6R, the ectodomain of IL-11R is subjected to N-linked glycosylation. However, in contrast to IL-6R, glycosylation at Asn-194 seems to be essential for the transport to the plasma membrane and substitution of Asn-194 with alanine resulted in predominant localization to the ER (Agthe et al., 2018b).

### Aberrant Trafficking of Alpha Receptors Due to Disease-Associated Mutations

Recently, inactivating mutations of IL-6R were identified in two patients suffering from immunodeficiency and an abnormal inflammatory response, associated with eosinophilia and elevated IgE levels (Spencer et al., 2019). Both variants impaired IL-6 signaling while signaling of other IL-6 family cytokines was intact. While one of these variants did not integrate into the plasma membrane due to a premature stop codon, the IL-6R I279N substitution (Figure 2B) resulted in predominant intracellular localization due to impaired trafficking to the surface. It is possible that this variant is entrapped in the ER due to folding defects in the extracellular domain.

Also in IL-11R, loss-of-function mutations (Figure 2B) were identified in patients and found to be associated with craniosynostis, a juvenile disease that causes premature closure of skull sutures (Nieminen et al., 2011; Keupp et al., 2013). Some of these mutations were shown to cause impaired IL-11R trafficking to the plasma membrane thereby impairing cellular susceptibility to IL-11 (Agthe et al., 2018a). Albeit the detailed mechanism of retention has been addressed, it is likely that folding defects in the IL-11R ectodomain result in prolonged association with the ER quality control, resulting in the observed predominant localization to the ER.

Also in *LIFR* and *OSMR* loss-of-function mutations were identified. While *LIFR* mutations were associated with Stüve-Wiedemann syndrome (Dagoneau et al., 2004), a rare disease characterized by skeletal dysplasia, mutations in *OSMR* were found in patients with primary localized cutaneous amyloidosis (Arita et al., 2008). However, whether these variants have an impact on receptor trafficking has not been addressed yet.

### Mechanisms of Internalization

**FIGURE 3:**
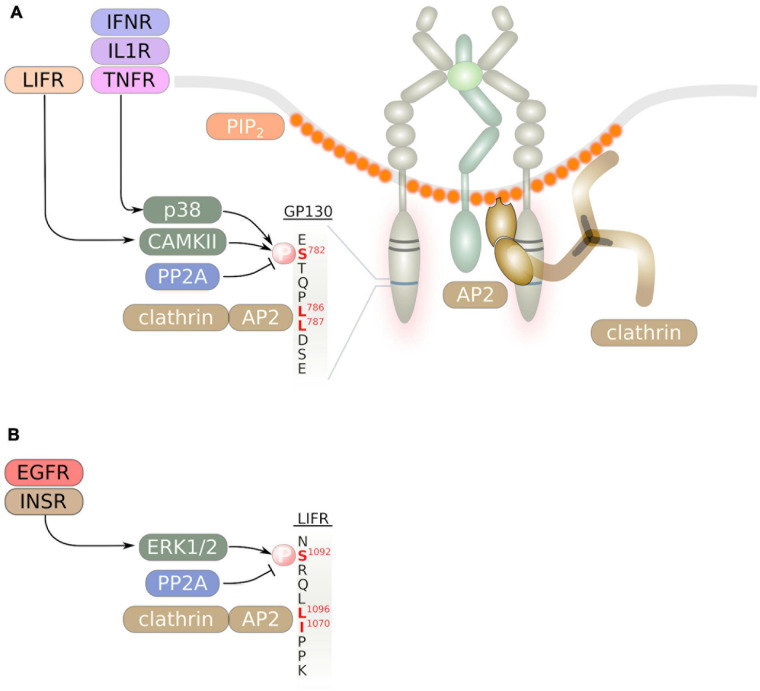
Internalization of IL-6 family signal transducing cytokine receptors occurs via a di-leucin motif. Binding of the adaptor protein complex AP2 to this di-leucin motif and to PIP_2_ at the inner leaflet of endocytosis-prone membrane areas initiates formation of clathrin-coated vesicles. The di-leucin motifs of GP130 **(A)** and the LIFR **(B)** are shown. Serine phosphorylation in close vicinity to the di-leucin motif is mediated by cytoplasmic serine/threonine kinases and enhances receptor internalization.

Engulfment into clathrin-coated vesicles is the most common and probably best studied way of receptor internalization and involves the recruitment of clathrin to heterotrimeric adaptor protein (AP) complexes. The reader is referred to recent reviews for further details (Le Roy and Wrana, 2005; Edeling et al., 2006; Briant et al., 2020; Homma et al., 2021). Four different adaptor protein complexes AP1-AP4 that promote formation of clathrin-coated vesicles were identified. AP2 was shown to interact with the cargo either via a YxxΦ motif or a [D/E]XXXL[L/I] ‘acidic di-leucine’ motif. It initially binds to membrane sites with accumulated phosphatidylinositol (4,5)-bisphosphate (PIP_2_) at the inner leaflet of the membrane (Figure 3A) which leads to a conformational change exposing the cargo binding site (Owen et al., 2004; Edeling et al., 2006). PIP_2_ also promotes binding of the GTPase dynamin to the plasma membrane that catalyzes pinching off of the endocytic vesicles (Briant et al., 2020).

The C-terminus of GP130 contains a STQPL^786^L^787^ di-leucine motif (Figure 3A), that was shown to be essential for efficient internalization of GP130. Substitution of both leucine residues with alanine largely impaired internalization and strongly delayed GP130 degradation (Dittrich et al., 1996). Interestingly, the GP130 di-leucine motif was also shown to mediate basolateral sorting of GP130 in polarized MDCK cells. C-terminal truncation of GP130 led to apical sorting of GP130 (Doumanov et al., 2006). However, little is known if this sorting mechanism also applies *in vivo* and in human cells. A similar SRQFL^1069^I^1070^ di-leucine motif was identified in the C-terminus of LIFR (Figure 3B) that is essential for LIFR internalization (Thiel et al., 1999). GP130 was shown to be constitutively associated with the adaptor protein complex AP-2 (Figure 3A; Thiel et al., 1998). Consequently, internalization of GP130 can be blocked by inhibitors of clathrin or via inhibition of dynamin (Schmidt-Arras et al., 2014; Martinez-Fabregas et al., 2019; Flynn et al., 2021). Consistent with the finding that AP-2 is constitutively associated with GP130, internalization of GP130 was demonstrated to occur independent of ligand-binding or JAK activity (Thiel et al., 1998; Flynn et al., 2021).

A recent report suggested that GP130 internalization kinetics depend on the half live of signaling receptor complexes. In this study, the authors generated IL-6 variants that bind to GP130 independently of IL-6R and with differential affinity toward GP130. Using these variants, the authors demonstrate that the half-life of cytokine/receptor complexes depend on the affinity of the ligand toward GP130. Furthermore, they suggest that long lived cytokine/receptor complexes display enhanced internalization kinetics with localization to EEA1-decorated early endosomes and enhanced degradation kinetics in RPE1 and HeLa cells. The highest internalization rates in this study were found for the HyperIL-6/GP130 complex. This is in stark contrast to previous findings, where HyperIL-6 was found to induce a stable long-lived receptor complex at the plasma membrane of HepG2 cells that displayed reduced internalization kinetics as compared to the IL-6/IL-6R/GP130 complex (Peters et al., 1998). Furthermore, one of the engineered IL-6 variants that had similar GP130 binding affinities as HyperIL-6 displayed a significantly reduced internalization kinetic as compared to HyperIL-6 (Martinez-Fabregas et al., 2019). It is therefore possible that GP130 internalization rates are cell type-dependent and dependent on the type of receptor complex formation.

Albeit Janus kinase activity and therefore GP130 tyrosine phosphorylation is dispensable for GP130 internalization, phosphorylation of Ser-782 that lies in vicinity of the di-leucine motif was demonstrated to enhance GP130 internalization and degradation. A S782A substitution resulted in enhanced cell surface localization of GP130 (Gibson et al., 2000). While LIF stimulation induced GP130 Ser-782 phosphorylation by calmodulin-dependent kinase type (CAMK) II (Gibson et al., 2000, 2005), Ser-782 becomes phosphorylated via p38-activated MAPK-activated protein kinase (MAPKAPK) 2 downstream of the pro-inflammatory cytokines IL-1β, TNF and IFNγ (Radtke et al., 2010; Zha et al., 2017). As a consequence, GP130 downstream signaling is abrogated. Cross-phosphorylation of Ser-782 in GP130 downstream of pro-inflammatory cytokines and subsequent enhanced internalization of GP130 therefore represents a safe-guard mechanism to prevent exacerbated pro-inflammatory signaling.

Similarly, internalization of LIFR is enhanced via ERK1/2-dependent phosphorylation of Ser-1044 that lies upstream of the di-leucine motif (Blanchard et al., 2000). Accordingly, extracellular stimuli such as insulin and EGF enhanced LIFR internalization (Schiemann et al., 1995; Blanchard et al., 2000).

Phosphorylation of GP130 Ser-782 and potentially also of LIFR Ser-1044 is counterbalanced by the serine phosphatase protein phosphatase (PP) 2A and inhibition of PP2A by okadaic acid enhanced degradation of GP130 (Mitsuhashi et al., 2005).

Interestingly, mutations in *IL6ST* that lead to ligand-independent GP130 activation resulted in differential localization of GP130 to early endosomes, depending on the type of mutation (Schmidt-Arras et al., 2014). Albeit Ser-782 phosphorylation of these variants was not investigated in this study, it is possible that either the amplitude of downstream signaling or receptor ectodomain conformations modulate kinetics and routes of internalization.

Most recently, *IL6ST* loss-of-function mutations (Figure 2A) were identified in patients suffering from hyper-IgE syndrome (Béziat et al., 2020). These mutations resulted in truncated GP130 variants I719 frameshift (fs) and T761fs lacking STAT3 binding sites and the di-leucin motif. Consequently, these variants displayed largely enhanced plasma membrane localization due to impaired internalization. Interestingly, these GP130 variants exhibited a dominant negative effect over wildtype gp130 in particular on IL-6 and IL-11 signaling. Accordingly, these mutations appeared to be monoallelic in all patients in this study. The authors demonstrated that this effect was at least in part due to sequestration of α receptors. However, it is also plausible that heterodimerization of an inactive variant with wildtype GP130 prolongs the dwell time of wildtype GP130 at the plasma membrane therefore further impairing downstream signaling.

While internalization seems to occur ligand-independently, lysosomal localization of GP130 was enhanced by IL-6 in overexpressing HeLa cells (Flynn et al., 2021). Also in murine CD4^+^ and CD8^+^ T-cells, IL-6 stimulation reduced plasma membrane localization of GP130. Accordingly, GP130 was barely detectable in IL-6/sIL-6R double transgenic mice that exhibit constitutive GP130 signaling (Wang et al., 1998), indicating that GP130 phosphorylation and downstream signaling not only enhances internalization but also induces GP130 degradation.

Degradation of GP130 can be mediated by both, lysosome and proteasome. It was demonstrated that the E3 ubiquitin ligase CBL is recruited to GP130 via the associated and tyrosine-phosphorylated PTPase SHP-2. The subsequent trafficking of GP130 to early and then late endosomes is mediated by the sorting protein HGS/Hrs. This protein can engage the ESCRT-0 complex that has been shown to mediate sorting to the endosomal compartment (Vietri et al., 2020). GP130 is subsequently degraded in lysosomes. Deficiency in c-Cbl or HGS results in enhanced and prolonged IL-6 signaling (Tanaka et al., 2008). However, this report did not investigate ligand-independent internalization and degradation.

Independent of ligand-binding IL-6R and IL-11R are endocytosed via clathrin-coated vesicles (Monhasery et al., 2016). Beside internalization, α-receptors can be removed from the cell surface via limited proteolysis (Müllberg et al., 1993; Lokau et al., 2016). The ectodomain of both, IL-6R and IL-11R can be proteolytically processed primarily by membrane-bound proteases of the a disintegrin and metalloprotease (ADAM) family (Zunke and Rose-John, 2017). While this abrogates cytokine signaling in the donor cell, it enables trans-signalling of GP130 in a paracrine fashion on neighboring cells (Peters et al., 2000).

## Signal Transduction at Endomembranes

Under physiological conditions, signal transduction of cytokine receptors is initiated at the plasma membrane upon ligand binding. It has become evident that membrane compartmentalization contributes to the regulation of receptor activation. Lipid rafts are dynamic membrane microdomains that are enriched in cholesterol, sphingolipids and GPI-anchored proteins (Le Roy and Wrana, 2005). Due to its hydrophobic nature and its planar and rigid structure, cholesterol favors interaction with saturated lipids with polar headgroups such as sphingolipids yielding the formation of dynamic nanoscale lipid assemblies. Membrane proteins were shown to have sphingolipid binding motifs. It was therefore speculated that protein interaction with “raft lipids” facilitates assembly and functionalization of ordered membrane rafts. Clustering of membrane proteins such as GPI-anchored proteins were shown to promote larger cholesterol-containing spatial and temporal assemblies which are often stabilized by cortical actin (Lingwood and Simons, 2010).

CNTFR but not LIFR and GP130 was shown to reside in the plasma membrane inside lipid rafts in a neuroblastoma cell line. However, upon CNTF but not LIF-stimulation, both, GP130 and LIFR translocated to lipid rafts (Port et al., 2007). In multiple myeloma cells, GP130 was found to be constitutively bound to caveolin-1 that is associated with lipid rafts (Podar et al., 2003). Interestingly, STAT1 and STAT3 were also found to be pre-associated with lipid rafts (Sehgal et al., 2002). Disruption of lipid rafts by methyl-β-cyclodextrin abolished IL-6 induced STAT activation in these cells (Sehgal et al., 2002; Podar et al., 2003), while it did not impair CNTF- or LIF-induced STAT3 phosphorylation in neuroblastoma cells (Port et al., 2007). It can therefore be concluded that residency of GP130 within membrane microdomains and the associated downstream signaling depends on the type of the receptor complex and the cellular context. However, further research is warranted to clarify the mechanisms that regulate sorting of GP130 into different microdomains and its consequences for downstream signaling and biological outcome. Interestingly, also SOCS3 was found to directly bind to caveolin-1 and regulate its stability. Thereby caveolin-1 recruits SOCS3 independent of its SH2 domain. Surprisingly, genetic deficiency of caveolin-1 resulted in enhanced STAT3 phosphorylation upon IL-6 stimulation (Williams et al., 2018). Therefore, caveolin-1 mediates efficient feedback inhibition of STAT3 activation at the plasma membrane.

Signaling of receptor molecules does not terminate at the plasma membrane but can continue throughout the endocytic pathway. There is compelling evidence that receptor tyrosine kinases continue to signal from endomembranes, including the endosomal compartment (Schmidt-Arras and Böhmer, 2020). Also for the IL-6 family cytokine receptors an increasing number of studies demonstrate continuation of signaling from within the endosomal compartment (see below).

**FIGURE 4:**
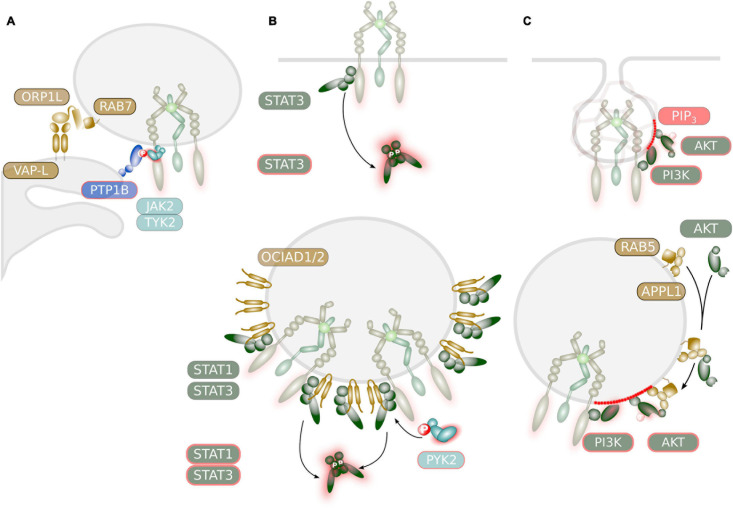
Potential mechanisms regulating signaling of IL-6 family cytokine receptors at endosomes. **(A)** ER-contact sites might regulate JAK activity. The ER-resident PTPase PTPN1/PTP1B was demonstrated to deactivate STAT signaling through dephosphorylation of JAK2 or TYK2. This might occur at ER-endosome contact sites as was demonstrated for the EGFR. These contact sites are mediated via interaction of the integral ER protein VAP-L and the endosomal associated protein ORP1L which is a RAB7 effector. **(B)** Selective and sustainable STAT activation at endosomal membranes. STAT proteins with high receptor affinity are readily activated at the plasma membrane. Activation of lower affinity STAT proteins might depend on endosomal localization of the receptor. STAT activation by IL-6 family cytokines at endosomes might be facilitated by the endosome-associated protein OCIAD1/ASRIJ and OCAID2. Recruitment of PYK2 to endosomes might prolong STAT activation as was demonstrated for the EGFR and HGFR/MET. **(C)** Compartment-specific AKT activation. The generation of PIP_3_ occurs in two waves: a first short peak at the plasma membrane and a second peak during clathrin-mediated endocytosis. Recruitment of PI3K isoforms to endosomes is mediated via RAB5. Activation of AKT by IL-6 family cytokine might be facilitated by the endosome-associated adaptor protein APPL1 that helps to recruit inactive AKT.

The notion that signal emission from endosomes involves ER-endosome contacts stems from the observation that dephosphorylation of several ligand-stimulated receptor tyrosine kinases such as the epidermal growth factor receptor (EGFR), c-Met and the granulocyte colony-stimulating factor receptor (G-CSFR) is mediated by the ER resident protein tyrosine phosphatase (PTP) PTPN1/PTP1B (Ostman and Böhmer, 2001). This has been in particular demonstrated for the EGFR, where internalized EGFR co-localized with PTP1B at ER-endosome contact sites (Haj et al., 2002; Eden et al., 2010). PTP1B was also identified as PTPase for JAK2 and Tyk2 (Myers et al., 2001) and as a regulator of leptin- or G-CSF-induced STAT3 activation (Cheng et al., 2002; Zabolotny et al., 2002; Palande et al., 2011). Downregulation of PTP1B expression resulted in enhanced downstream signaling of GP130, suggesting that inactivation of GP130 signaling also occurs at ER-endosome contact sites (Figure 4A; Fukada and Tonks, 2003). However, experimental evidence for this assumption is still lacking.

ER-endosome contact sites are established during the maturation of the endosome and it is thought that the majority of late endosomal (LE) vesicles are in contact with the ER (Raiborg et al., 2015). ER-endosome contact sites are established through interaction of the integral ER protein vesicle-associated protein (VAP) A with endosomal membrane-associated proteins. Among those, the cholesterol-sensing protein ORP1L is recruited to the late endosome via direct interaction with the small GTPase RAB7 (Figure 4A; Raiborg et al., 2015).

### Activation of STAT Proteins

Recruitment and activation of STAT proteins is a key signaling event in IL-6 family cytokine receptors (Heinrich et al., 2003; Rose-John, 2018) and plasma membrane recruitment of STAT3 to the activated IL-6 receptor complex has been observed (Shah et al., 2006).

However, there are multiple indications that STAT activation can occur from endosomes and might be initiated after cytokine receptor internalization. A small fraction of STAT3 proteins were found to be constitutively associated with early endosomes (Shah et al., 2006) and it is therefore conceivable that internalized receptors can activate STAT proteins in endosomes “en passant.” The endosomal proteins OCIA domain-containing protein (OCIAD) 1/ASRIJ and OCIAD2 are members of the ovarian carcinoma immunoreactive antigen (OCIA) protein family. Both, OCIAD1/ASRIJ and OCIAD2 directly interact with STAT3 at endosomal vesicles (Figure 4B; Sinha et al., 2013, 2018). While OCIAD1 facilitates STAT3 phosphorylation (Sinha et al., 2013) OCIAD2 is essential for STAT3 activation (Sinha et al., 2018).

Activation of STAT1 and 3 downstream of the receptor tyrosine kinases EGFR, platelet-derived growth factor receptor (PDGFR)β and hepatocyte growth factor receptor (HGFR/MET) was observed to occur exclusively from endosomes (Bild et al., 2002; Kermorgant and Parker, 2008; Sadowski et al., 2013; Parks and Ceresa, 2014; Jastrzębski et al., 2017). Interestingly signaling outcome for HGFR/MET differs depending on the localization of endosomal vesicles (Ménard et al., 2014), further suggesting that contact of endosomal vesicles to the ER modulates receptor signaling. Endosomal STAT3 activation by EGFR and HGFR/MET is further enhanced through the recruitment of the cytoplasmic tyrosine kinase PYK2 to early endosomes, representing a positive feedback loop to sustain endosomal STAT3 activation and to promote epithelial-to-mesenchymal transition (EMT) and therefore tumor invasiveness (Verma et al., 2015).

There is strong evidence that IL-6-mediated STAT3 phosphorylation requires endosomal localization of GP130. Upon IL-6 stimulation, a large fraction of STAT3 is recruited to endosomal vesicles (Xu et al., 2007; German et al., 2011) and inhibition of clathrin-mediated endocytosis impairs GP130-induced STAT3 phosphorylation (Xu et al., 2007; Schmidt-Arras et al., 2014). Also STAT3 activation through co-trafficking of IL-6/IL-6R/GP130 complexes in intracellular compartments of dendritic cells occurred at endosomes (Verboogen et al., 2018). Along this line, inhibition of PP2A by okadaic acid blunted STAT3 activation downstream of IL-6 stimulation and correlated with reduced internalization and proteasomal degradation of GP130 (Mitsuhashi et al., 2005). GP130 was also found to activate PYK2, which might contribute to sustained endosomal activation of STAT3 (Figure 4B; Schaeffer et al., 2001). In contrast, inhibition of clathrin-mediated endocytosis did not block OSM-induced STAT3 activation (Kermorgant and Parker, 2008).

Therefore, it is not absolutely evident if STAT proteins are primarily activated from the endosome or from the plasma membrane. There is evidence that receptor complex stability, receptor phosphorylation/dephosphorylation kinetics, and affinity of STATs toward phosphorylated receptor chains determines localization of STAT activation. As an example, while STAT3 activation by OSM occurred rapidly and independent of internalization, STAT3 phosphorylation downstream of HGFR/MET occurred exclusively in endosomes at a later time point after stimulation. This observation correlated with phospho-STAT3 signal strength: while OSM induces strong STAT3 phosphorylation, phospho-STAT3 signals induced HGFR/MET are rather weak (Kermorgant and Parker, 2008). Furthermore, the decision on which particular STAT protein becomes activated seems to be spatially regulated.

These observations might be explained in part by the affinity of STAT SH2 domains toward a phosphorylated tyrosine residue in the receptor complex. Due to the different nature of their SH2-domains, STAT proteins possess differential affinity for phosphorylated tyrosine residues at receptor molecules. While STAT3 can bind to multiple phosphotyrosine residues in GP130, the binding of STAT1 is more restricted (Heinrich et al., 2003). In addition, STAT1 and STAT3 bind with different affinities to phosphorylated GP130 (Wiederkehr-Adam et al., 2003). As a consequence, STAT1 and STAT3 compete in particular for phosphorylated Tyr-905 and to a lower extent for phosphorylated Tyr-915 in GP130 (Heinrich et al., 2003; Martinez-Fabregas et al., 2019).

A very recent report used engineered IL-6 variants with variable affinity for GP130 that where independent of IL-6R binding. The affinity of ligands correlated with receptor complex dwell times and with internalization rates. As a consequence, short lived receptor complexes induced a high phospho-STAT3 to phospho-STAT1 ratio. Activation of STAT1 was lowered in intermediate affinity receptor complexes but not low or high affinity complexes, when clathrin-mediated endocytosis was impaired (Martinez-Fabregas et al., 2019). This suggests that internalization of lower affinity complexes enhances ligand-receptor dwell time and enables the low-affinity binder STAT1 to get activated at endosomes. Unfortunately, the impact of internalization on IL-6- or HyperIL-6-mediated STAT1 phosphorylation was not addressed in this study.

### Activation of the Ras/MAPK Pathway

Similar to receptor tyrosine kinases, upon ligand-stimulation at the plasma membrane, cytokine receptors induce several signal transduction modules including the activation of the small membrane-bound GTPase Ras and downstream activation of the mitogen activated protein kinase (MAPK) cascade.

There has been agreement that activation of the small GTPase RAS occurs at the plasma membrane. However, there is a growing number of reports demonstrating that among the four different RAS isoforms N-RAS, H-RAS, KRAS4A and K-RAS4B, N- and H-RAS are also localized to endomembranes, including the endosomes (Fehrenbacher et al., 2009). Activation of RAS isoforms at endomembranes can vary in amplitude (Aran and Prior, 2013) and have different biological outcomes (Daniels et al., 2006; Matallanas et al., 2006). As an example, activation of ERK downstream of EGFR activation initiates at the plasma membrane but continues in endosomes, where the signal strength is even increased (Anastasi et al., 2013).

IL-6 family receptors activate RAS and MAPK cascade by two different mechanisms. Phosphorylation of Tyr-759 in GP130 and Tyr-974 in LIFR, respectively, leads to the recruitment of tyrosine-protein phosphatase non-receptor type (PTPN) 11/SHP2. Subsequent phosphorylation of PTPN11/SHP2 creates a binding site for GRB2/SOS and subsequent immediate activation of RAS/MAPK pathway (Schiemann et al., 1997). In contrast, phosphorylation of Tyr-861 in OSMR recruits the adaptor protein SHC that gets phosphorylated and bound to GRB2/SOS (Hermanns et al., 2000). PTPN11/SHP2 can dephosphorylate GP130 (Lehmann et al., 2003), however its role as feedback inhibitor is under debate (Dittrich et al., 2012). However, GP130 Y759F substitution or genetic deficiency of PTPN11/SHP2 results in hyperactivation of STAT3 (Tebbutt et al., 2002; Jenkins et al., 2005; Bard-Chapeau et al., 2011). Interestingly, inactivation of SHP2 catalytic activity by NADPH oxidase (NOX) 1/4-induced ROS production downstream of the PDGFR occurred on early endosomes. While it is known that GP130 can activate the small GTPase Rac1 (Arulanandam et al., 2010), little is known if this also leads to NOX activation and ROS production.

Inhibition of dynamin-mediated internalization enhanced ERK phosphorylation after short term stimulation with IL-6 (Schmidt-Arras et al., 2014), indicating that endocytosis blunts the initial phase of GP130-mediated RAS/MAPK activation at the plasma membrane. Sustained RAS/MAPK activation downstream of GP130 needs the recruitment of the multi-site docking protein GAB1 to GP130 via PTPN11/SHP2 (Bongartz et al., 2019). Interestingly, sustained RAS/MAPK activation downstream of the EGFR is also mediated via GAB1 and recruitment of GAB1 to EGFR occurred at early endosomes (Kostenko et al., 2006). It is therefore likely, that sustained RAS/MAPK activation via GAB1 downstream of GP130 also occurs at endosomes. Further studies are warranted to dissect GP130-dependent spatial RAS/MAPK activation, resulting differences in signal quality and its biological consequences.

### The PI3Kinase Pathway

Beside STAT protein activation, activity of phosphoinositide-3 kinase (PI3K) and phosphorylation of phosphoinositol 4,5,-bisphosphate (PIP_2_) to generate the second messenger phosphatidylinositol 3,4,5,-trisphosphate (PIP_3_) is important for the biological effects of IL-6 type cytokines, in particular GP130. As such, PI3K was shown to be essential for GP130-induced pro-inflammatory signaling in endothelial cells (Morris et al., 2008; Suzuki et al., 2011; Zegeye et al., 2018), or tumorigenesis (Heinrich et al., 2003; Thiem et al., 2013; Werner-Klein et al., 2020).

It is common knowledge that generation of PIP_3_ occurs during clathrin-mediated receptor endocytosis due to RAB5-mediated recruitment of PI3K (Zerial and McBride, 2001; Sato et al., 2003). Generation of PIP_3_ occurs in two waves due to differential recruitment of PIP3 phosphatases to endocytic vesicles. Recruitment of PI3K to the plasma membrane induces the occurrence of a short peak of PIP_3_ accumulation, which is rapidly terminated by the PIP_3_ phosphatase SHIP2 just before the clathrin-coated endocytic vesicles pinch off the membrane (Figure 4C). Upon dissociation of the clathrin coat, receptor-associated PI3K induces a second peak of PIP_3_. Endocytic vesicles can fuse with a myriad of different types of endosomal vesicles. One type of vesicles is decorated with the adaptor protein, phosphotyrosine interaction, PH domain, and leucine zipper containing (APPL) 1 (Naguib, 2016). APPL1 is a RAB5 effector protein that directly interacts with protein kinase B (PKB)/AKT (Mitsuuchi et al., 1999) and determines its substrate specificity (Schenck et al., 2008). As such, APPL1 was demonstrated to facilitate AKT activation downstream of the EGFR (Jones et al., 2006) and the insulin receptor (Saito et al., 2007) on endosomal vesicles (Figure 4C). While recruitment of class I PI3Kα to activated receptor tyrosine kinases at endomembranes is mediated via microtubule-associated protein (MAP) 4 and microtubules (Thapa et al., 2020), the liver-specific class II PI3K-C2γ directly interacts with RAB5 to mediate AKT activation downstream of hepatic insulin signaling (Braccini et al., 2015). Albeit APPL1 strongly interacts with PI3K in thymic T cells, APPL1 is dispensable for AKT activation in these cells (Tan et al., 2010, 2016). In contrast, HGFR/MET-induced AKT activation in fibroblasts depends on APPL1 (Tan et al., 2016).

Unfortunately, only little is known about spatial regulation of PI3K signaling upon IL-6 family receptor activation. Given its dominant expression in T-cells and hepatocytes, it is possible that GP130 employs RAB5 and APPL1 for PI3K activation and endosomal membranes. There are indeed some indications that GP130 induces PIP_3_-dependent signaling from endosomes. In response to IL-6 stimulation, recruitment of PI3K regulatory subunit p85 to GP130 occurs via phosphorylated GAB1 (Heinrich et al., 2003). As outlined above, it is likely that GAB1 is recruited to GP130 at endosomes. Furthermore, activation of the mammalian target of rapamycin complex (mTORC) by GP130 requires PI3K activity (Thiem et al., 2013) and mTORC1 and 2 were found to be associated with endosomal vesicles and the lysosome (Ögmundsdóttir et al., 2012; Ebner et al., 2017; Marat et al., 2017). While accumulation of plasma membrane PIP_3_ is linked to cellular migration (Devreotes and Horwitz, 2015; Yan et al., 2021), was mTORC activation shown to mediate autophagy and survival (Kim and Guan, 2019). It is therefore conceivable that differential spatial activation of PI3K downstream of GP130 has differential biological outcomes. Hence, detailed temporal and spatial analysis of GP130-induced PI3K signaling is highly warranted, in particular dependent on the cellular context.

## Conclusion and Perspectives

The concept of spatial regulation of intracellular signaling has only recently emerged. While data on spatial signaling of receptor tyrosine kinases is increasing, only little is known about compartmentalization of cytokine signaling, in particular for IL-6 family cytokine receptors and warrants further investigation. Given the fact that biological outcome is dependent on compartment-specific signaling (Daniels et al., 2006; Matallanas et al., 2006; Martinez-Fabregas et al., 2019), a more detailed knowledge on the mechanisms of spatial regulation of cytokine receptor signaling would open up a new avenue for therapeutics design. In this respect, it is not only interesting to answer the question e.g., on the spatial activation of STAT proteins but to investigate if compartment-specific signals alter signal quality. As an example, kinetics of STAT activation might not only determine nuclear shuttling rates but as a direct consequence could alter on-off rates on STAT-responsive promoters and therefore regulate expression of STAT-responsive genes in a specific manner. A recent report has demonstrated that fine-tuning of STAT3 resident time at STAT-responsive genes indeed alters gene expression (Martinez-Fabregas et al., 2020). The design of cytokine variants that favor particular compartment-specific receptor signaling would be an elegant way for the design of future therapeutics.

Targeting of the artificial LGP130 variant to different subcellular compartments would allow to identify GP130-initiated, compartment-specific signaling pathways independent of ligand stimulation and without the need of receptor endocytosis. When inserted into the ROSA26 locus, expression of these variants could be initiated in a Cre-/Flp-recombinase-dependent and cell type-specific manner. This would allow analysis of the impact of compartment-specific signaling modules on biological outcomes of a particular cell type.

In combination with the aforementioned novel designer cytokines we would be able to fine-tune GP130 signaling in preclinical disease models and open up the avenue for novel therapeutics.

## Author Contributions

DS-A and SR-J conceived, wrote, and edited the manuscript. DS-A generated artwork. Both authors contributed to the article and approved the submitted version.

## Conflict of Interest

The authors declare that the research was conducted in the absence of any commercial or financial relationships that could be construed as a potential conflict of interest.
